# Extracellular matrix marker LAMC2 targets ZEB1 to promote TNBC malignancy via up-regulating CD44/STAT3 signaling pathway

**DOI:** 10.1186/s10020-024-00827-6

**Published:** 2024-05-17

**Authors:** Ding Wang, Kailibinuer Keyoumu, Rongji Yu, Doudou Wen, Hao Jiang, Xinchun Liu, Xiaotang Di, Shubing Zhang

**Affiliations:** 1 Department of Cell Biology, School of Life Sciences, Central South University, Changsha, 410013 Hunan China; 2 The Third Xiangya Hospital, Central South University, Changsha, 410013 Hunan China; 3 Department of Biomedical Informatics, School of Life Sciences, Central South University, Changsha, 410013 Hunan China; 4 The Affiliated Changsha Central Hospital, Hengyang Medical School, University of South China, Changsha, 410000 Hunan China; 5 Hunan Key Laboratory of Animal Models for Human Diseases, Central South University, Changsha, 410013 Hunan China

**Keywords:** Triple negative breast cancer, LAMC2, Migration, EMT, ZEB1, CD44, STAT3

## Abstract

**Background:**

Triple negative breast cancer (TNBC) is a heterogeneous and aggressive disease characterized by a high risk of mortality and poor prognosis. It has been reported that Laminin γ2 (LAMC2) is highly expressed in a variety of tumors, and its high expression is correlated with cancer development and progression. However, the function and mechanism by which LAMC2 influences TNBC remain unclear.

**Methods:**

Kaplan–Meier survival analysis and Immunohistochemical (IHC) staining were used to examine the expression level of LAMC2 in TNBC. Subsequently, cell viability assay, wound healing and transwell assay were performed to detect the function of LAMC2 in cell proliferation and migration. A xenograft mouse model was used to assess tumorigenic function of LAMC2 in vivo*.* Luciferase reporter assay and western blot were performed to unravel the underlying mechanism.

**Results:**

In this study, we found that higher expression of LAMC2 significantly correlated with poor survival in the TNBC cohort. Functional characterization showed that LAMC2 promoted cell proliferation and migration capacity of TNBC cell lines via up-regulating CD44. Moreover, LAMC2 exerted oncogenic roles in TNBC through modulating the expression of epithelial-mesenchymal transition (EMT) markers. Luciferase reporter assay verified that LAMC2 targeted ZEB1 to promote its transcription. Interestingly, LAMC2 regulated cell migration in TNBC via STAT3 signaling pathway.

**Conclusion:**

LAMC2 targeted ZEB1 via activating CD44/STAT3 signaling pathway to promote TNBC proliferation and migration, suggesting that LAMC2 could be a potential therapeutic target in TNBC patients.

**Supplementary Information:**

The online version contains supplementary material available at 10.1186/s10020-024-00827-6.

## Introduction

Triple negative breast cancer (TNBC) is one of the most aggressive and lethal cancers, which is projected to become the leading cause of cancer-related mortality in women (Siegel et al. 2022). Due to significantly higher rates of metastasis and recurrence, the 5-year survival rates for TNBC remain less than 30% (Early Breast Cancer Trialists' Collaborative Group 2019). Because of the lack of well-defined molecular targets, surgical resection and chemotherapy are currently still the major clinical therapies for TNBC patients (Giaquinto et al. 2022). Therefore, it is essential to explore more effective biomarkers for the early diagnosis and treatment of TNBC.

Laminin γ2 (LAMC2), a subunit of the heterotrimeric glycoprotein laminin-332, is recognized as an important component of epithelial basement membranes and regulates cell adhesion, differentiation and migration (Rousselle and Scoazec 2020). Accumulating evidence in recent years has revealed that LAMC2 is highly expressed and acts as an oncogenic gene in a variety of cancers, suggesting that LAMC2 might be a potential diagnostic and prognostic biomarker in patients with cancer (Daisuke et al. 2021; Liu et al. 2021). Notably, Sato et al*.* found that LAMC2 directly interacted with CD44 to stimulate migration of breast cancer cells (Sato et al. 2015). However, the exact signaling pathway regulated by LAMC2/CD44 in TNBC is still not fully understood.

CD44 is a non-kinase cell surface transmembrane glycoprotein and identified as a breast cancer stem cell (CSC) marker (Zhang et al. 2019). Yang et al*.* found that CD44 is a direct target of miR-143 to inhibit the stemness of breast cancer cells (Yang et al. 2016). Interestingly, CD44 undergoes alternative splicing, resulting in the standard (CD44s) and variant (CD44v) isoforms and CD44s is currently the dominant isoform expressed in breast CSCs (Zhang et al. 2019; Mesrati et al. 2021). Additionally, many studies have indicated that CD44 can regulate the EMT signaling pathway and epithelial plasticity in breast cancer and other cancers (Brown et al. 2011; Chen et al. 2018; Yang et al. 2019). For instance, TGF-β1 induced CD44 alternative splicing to promote EMT and stemness in prostate cancer (Chen et al. 2021). Moreover, Marotta et al*.* found that the JAK2/STAT3 signaling pathway is required for the growth of CD44^+^CD24^–^ stem cell-like breast cancer cells (Marotta et al. 2011; Stevens et al. 2023). High expression of JAK/STAT3 was associated with poor prognosis in TNBC (Morrow et al. 2023). Inhibiting the downstream signaling of JAK/STAT3 pathway leads to the induction of cell apoptosis, which can be used to discover anticancer drug.

In the present study, for the first time, we explored the underlying mechanisms of LAMC2 in TNBC. We found that high expression of LAMC2 is associated with poor prognosis in TNBC patients. Over-expression of LAMC2 in TNBC cells promoted cell proliferation and migration preferentially through up-regulating CD44. Moreover, LAMC2 exerted oncogenic roles in TNBC cellular growth and migration via the EMT marker gene ZEB1. Furthermore, LAMC2 activated the STAT3/ZEB1 signaling pathway to promote cell migration in TNBC cells. Overall, we identified LAMC2 as a new prognostic biomarker and a potential therapeutic target in TNBC.

## Materials and methods

### Cell culture and transfection

MDA-MB-231 and BT549 cells were cultured in DMEM (SH30243.01, hyclone, USA) containing 10% fetal bovine serum at 37 °C and 5% CO_2_. According to the manufacturer's instructions, 3–5 μL Hieff TransTM Liposomal Transfection Reagent (Cat# 40802ES03, Yeasen, China) and 3–6 μg plasmid were used to transfect MDA-MB-231 and BT549 cells. qPCR and western blot were performed to confirm transfection efficiency.

### Patients and tissue samples

BC and adjacent normal breast tissue samples were obtained from 6 breast cancer patients who had undergone primary surgical resection at the second Xiangya Hospital, Central South University between June 2021 and December 2023. TNBC and non-TNBC tissue samples were obtained from 26 breast cancer patients who had undergone primary surgical resection at the third Xiangya Hospital, Central South University between July 2016 and March 2018. All experiments performed in studies involving human participants were approved by the Ethical Committee of the School of Life Sciences, Central South University (No. 2020-1-34). All patients have signed the consent form.

### Plasmid and stable cell line construction

The LAMC2 shRNA sequences (http://crispor.tefor.net) were cloned into the pLKO.1 vector using the following primers: shRNA1-LAMC2-F: CCGGGCAGGCACTTACTACTAATAACTCGAGTTATTAGTAGTAAGTGCCTGCTTTTTG,shRNA1-LAMC2-R:AATTCAAAAAGCAGGCACTTACTACTAATAACTCGAGTTATTAGTAGTAAGTGCCTGC,shRNA2-LAMC2-F:CCGGCCACTCCTGGAACTCATCTTTCTCGAGAAAGATGAGTTCCAGGAGTGGTTTTTG,shRNA2-LAMC2-R:AATTCAAAAACCACTCCTGGAACTCATCTTTCTCGAGAAAGATGAGTTCCAGGAGTGG.The pLKO.1 plasmid with the target sequence was digested with EcoRI and AgeI (Cat#:FD0834, Thermo Scientific, China). The LAMC2 gene sequences (https://www.ncbi.nlm.nih.gov) were cloned into the pCDH-CD513B-1-PHB vector using the following primers: LAMC2-F: cgGGATCCatgcctgcgctctggctg, LAMC2-R: cgGAATTCtcacatacccatcagatg. The pCDH-CD513B-1-PHB plasmid with the target sequence was digested with EcoRI and BamHI (Cat #FD0834, Thermo Scientific, China). The constructed plasmid was transfected into HEK293T cells by the Liposome transfection method. Puromycin (0.5 μg/mL for MDA-MB-231, 2 μg/mL for BT549) was added for 3 days to obtain a stable over-expression cell line. The effect of over-expression was detected by qPCR.

### CCK8 assay

Cells were digested with trypsin and resuspended in 3 mL complete medium. The cell density was adjusted to 3 × 10^4^/mL, and 200 μL cell suspension was inoculated into 96-well plates. After 1 day, 3 days, 5 days, the supernatant was discarded and CCK-8 (Cat# C0005, Target Mol, USA) working fluid (cck8:cell suspension = 1:10) was added to 96-well plates and incubated at 37 °C for 4 h. The OD value at 450 nm was determined using a microplate reader (EnSpire 2300, PerkinElmer, USA). Each group was repeated three times.

### Transwell assay

Cells were digested with trypsin, resuspended in 3 mL medium, and diluted to a density of 2 × 10^4^/mL. A total of 100 μL cell suspension was inoculated into the upper chamber of a Transwell chamber without FBS, and 700 μL medium with 10% FBS was added to the lower chamber. For invasion assay, the chamber was pre-coated with Matrigel (Ref 356234, Corning, USA). The rest of the procedures were performed in the same way as the migration assay. 48 h later, the medium was discarded, and the cells were washed with PBS three times, fixed with 4% paraformaldehyde for 20 min, and stained with 0.1% crystal violet for 20 min. The images of stained cells on the lower side were captured by microscope.

### Wound healing assay

Cells were cultured in the 6-well plate until the cells covered the entire surface. A 100 µL pipette tip was used to scrape off the cells. Then the cells were washed three times with PBS and added serum-free medium. Subsequently, the cells were cultured in a 5% CO_2_ incubator at 37 °C. Images of the cells were taken using an inverted microscope at 0, 24, 48 h.

### Immunohistochemistry

The tissue sections were washed in 0.01M PBS for 3 × 5 min, heated with citric acid antigen repair solution at 98 °C for 30 min, and then incubated in 0.3% H_2_O_2_ for 30 min. They were subsequently blocked in 0.01M PBS supplemented with 0.3% Triton X-100 and 5% normal goat serum for 1 h, and then incubated with LAMC2 antibody at 4 °C overnight. After a short wash in 0.01M PBS, goat anti-rabbit IgG coupled with horseradish peroxidase was incubated in 0.01M PBS for 2 h and then treated with 0.03% DAB in 0.05M Tris–HCl (pH 7.6). All sections were counterstained with hematoxylin.

### EdU assay

Cells were cultured in a 96-well plate until they reached 70% confluency. The Click EdU-555 Cell proliferation detection kit (Cat# E2051, APPLYGEN, China) was used. Two hours before the experiment, 2 × EDU working liquid (20 μmol/L) was prepared and added to the incubator for preheating. Then, the experimental procedure was carried according to the manufacturer’s instructions. The nuclei were stained with Hoechst 33342 staining solution. Finally, they were washed twice with PBS buffer solution and examined by fluorescence microscope.

### RNA extraction and quantitative PCR

Total RNA was isolated from cells using the RNA simple Total RNA Kit (Cat#:DP419, TIANGEN, China) 0.1 μg of total RNA was used with the FastKing gDNA Dispelling RT SuperMix Kit (Cat#:KR118, TIANGEN, China) to synthetic cDNA. Real-time quantitative PCR (qRT-PCR) reactions were performed using the qRT-PCR Kit (Cat#:11201ES03, YEASEN, China). The sequences of qRT-PCR primers were list as follows (LAMC2 forward Primer: GACAAACTGGTAATGGATTCCGC, LAMC2 reverse Primer: TTCTCTGTGCCGGTAAAAGCC, ZEB1 forward Primer: GATGATGAATGCGAGTCAGATGC, ZEB1 reverse Primer: ACAGCAGTGTCTTGTTGTTGT, GAPDH forward Primer: GGAGCGAGATCCCTCCAAAAT, GAPDH reverse Primer: GGCTGTTGTCATACTTCTCATGG, β-catenin forward Primer: AAAGCGGCTGTTAGTCACTGG, β-catenin reverse Primer: CGAGTCATTGCATACTGTCCAT, Snail forward Primer: TCGGAAGCCTAACTACAGCGA, Snail reverse Primer: AGATGAGCATTGGCAGCGAG, slug forward Primer: CGAACTGGACACACATACAGTG, slug reverse Primer: CTGAGGATCTCTGGTTGTGGT, Twist forward Primer: GTCCGCAGTCTTACGAGGAG, Twist reverse Primer: GCTTGAGGGTCTGAATCTTGCT, ZEB2 forward Primer: CAAGAGGCGCAAACAAGCC, ZEB2 reverse Primer: GGTTGGCAATACCGTCATCC). The relative mRNA expression of these target genes was normalized using the endogenous GAPDH as a control and quantified using the 2^−ΔΔCt^ method.

### Western blot

Protein lysates were extracted using RIPA buffer [0.1% SDS, 1% NP-40, 0.5% sodium deoxycholate, 50 mM Tris (pH = 7.4)] supplemented with phosphatase (Cat:GK10011,GLPBIO,USA) and protease inhibitors (Cat:GK10014,GLPBIO,USA). Protein concentration was quantified by the BCA reagent (Cat#:BCA02, DingGuo, China). Proteins, separated by SDS-PAGE and transferred onto PVDF membranes, were probed with antibodies. Horseradish peroxidase-conjugated secondary antibodies were purchased from Cell Signaling Technology. The antibodies were listed as follows: LAMC2 (Cat#:sc-28330, Santa Cruz, USA), FLAG (Cat#:F1804, USA), GAPDH (Cat#:AP0066, Bioworld, USA), STAT3 (Cat#79D7, Cell signaling, USA), p-STAT3 (Cat#D3A7, Cell signaling, USA), Vimentin (Cat#D21H3, Cell signaling, USA), ZEB1 (Cat#E2G6Y, Cell signaling, USA), β-catenin (Cat#9562s, Cell signaling, USA), Snail (Cat#3879s, Cell signaling, USA).

### In vivo tumorigenesis

The nude mice were maintained in specific pathogen-free environments. LAMC2 over-expressing cells and controls were injected subcutaneously into 5-week-old male nude mice (five for each treatment). Tumor volumes were calculated using the formula length × (width)^2^/2. All animal experiments were performed in accordance with the guidelines from the Department of Laboratory Animals, Central South University.

### TCGA data analysis

The gene expression data from TCGA-BRCA were analyzed using the online tool GEPIA (http://gepia.cancer-pku.cn/) to obtain the correlation plot between LAMC2 and ZEB1.

### Luciferase assay

The HEK293T cells were evenly distributed into a 24-well plate, and the ZEB1 promoter along with the expression vector for Luciferase (Cat#1500, Promega, USA) were transfected into the cells using liposomes. Two days later, the lysed cells were assayed for luciferase activity using a Sirius single-tube chemiluminescence detector.

### Statistic analysis

Statistic analysis was performed using Prism 8.0 (GraphPad, San Diego, CA). An unpaired two-sided student *t-test* was used to calculate significance unless stated otherwise. When more than two groups were assessed, data were analyzed by one-way ANOVA. Significance was assumed to be reached at P < 0.05. *P < 0.05, **P < 0.01, ***P < 0.001. NS, not significant. All data were presented as mean ± SD.

## Results

### LAMC2 was identified to be highly increased in TNBC and associated with poor prognosis of TNBC patients

**Fig. 1:**
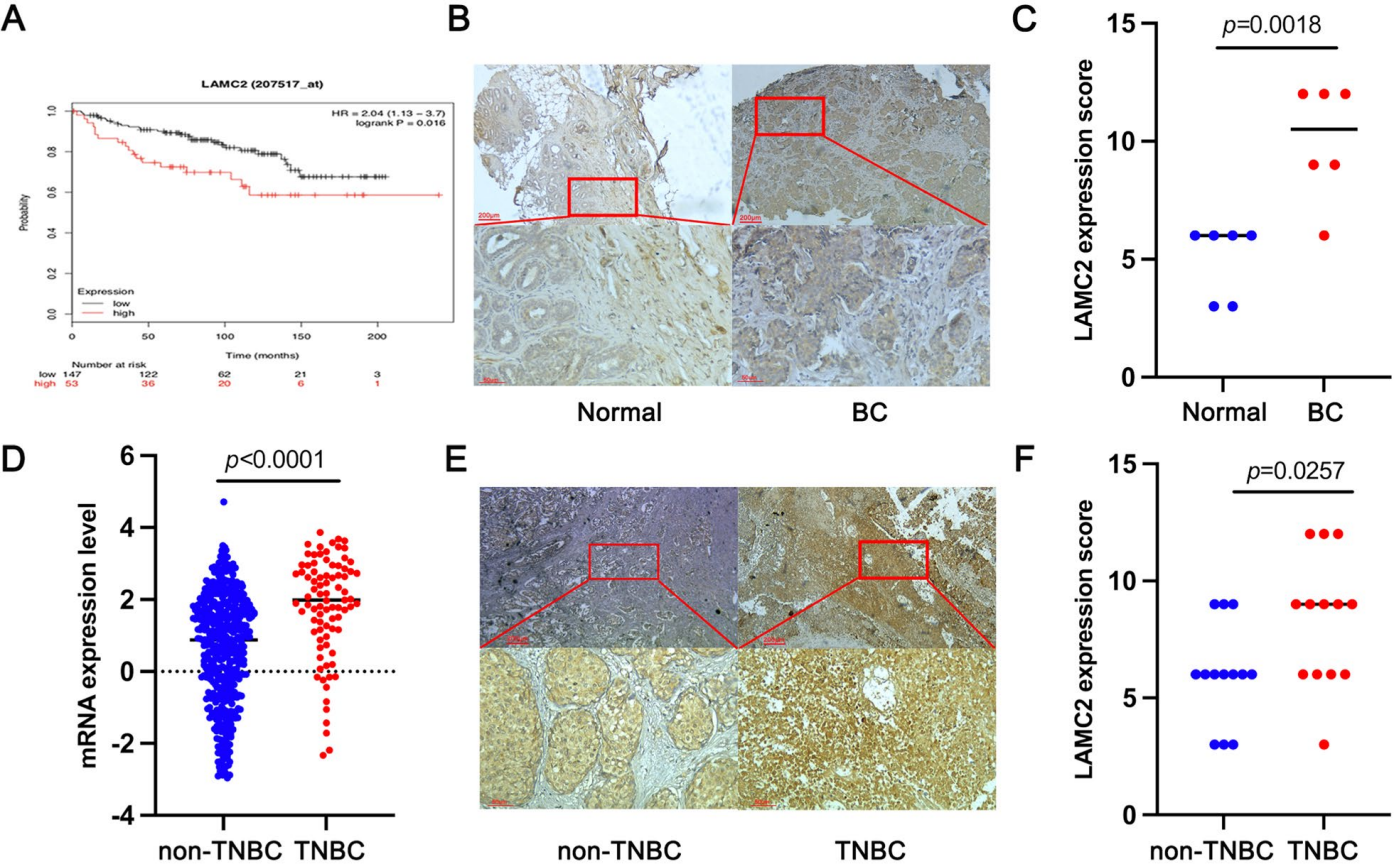
LAMC2 expression was highly increased in TNBC and associated with a poor prognosis for TNBC patients. **A** Kaplan–Meier overall survival analysis of patients with TNBC from The Cancer Genome Atlas database. **B**, **C** Representative IHC staining images and IHC scores of LAMC2 in BC tissues and adjacent normal breast tissues (n = 6) (original magnification ×20). **D** The mRNA expression levels of LAMC2 between TNBC tissues and non-TNBC tissues in the GEO dataset. **E**, **F** Representative IHC staining images and IHC scores of LAMC2 in TNBC tissues compared with non-TNBC tissues (n = 13) (original magnification ×20). Results were expressed as mean ± SD. Statistical analyses were analyzed using a Student’s *t*-test. *P* < 0.05 was considered as a significant difference

To investigate the function of LAMC2, a Kaplan–Meier survival analysis for overall survival was conducted. We found that high expression of LAMC2 was positively correlated with worse overall survival of BC patients (p = 0.016) (Fig. 1A). We then examined the expression level of LAMC2 in BC tissues and adjacent normal breast tissues using immunohistochemical (IHC) staining. Consistent with previous results, the data showed that LAMC2 was highly expressed in BC tissues compared to adjacent normal breast tissues (Fig. 1B, C). Many studies have shown that TNBC is a much more aggressive BC type due to faster spreading, fewer treatment options and worse prognosis. Therefore, LAMC2 expression was also examined in TNBC cells. Notably, we found that LAMC2 was more highly expressed in TNBC tissues compared to non-TNBC tissues (Fig. 1D). Likewise, IHC staining showed that LAMC2 was more highly expressed in TNBC tissues compared to non-TNBC tissues (Fig. 1E, F). Taken all together, these results suggested that LAMC2 expression was highly increased in TNBC and associated with poor prognosis of TNBC patients.

### LAMC2 silencing inhibited the proliferation and migration of TNBC cells in vitro

**Fig. 2:**
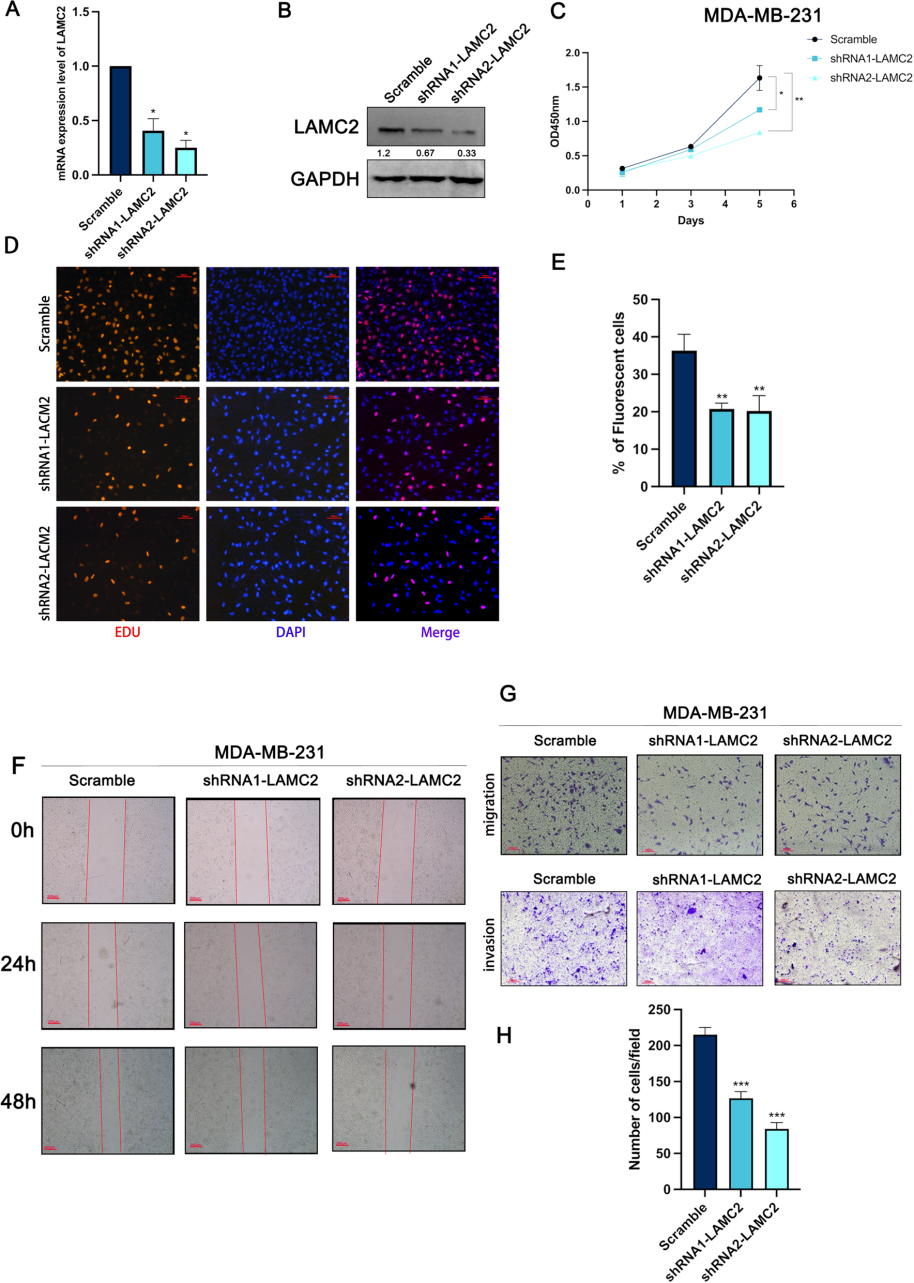
Knockdown of LAMC2 inhibited proliferation and migration in TNBC cells in vitro. **A**, **B** The expression of LAMC2 in MDA-MB-231 cell lines knockdown of LAMC2 was detected by qRT-PCR and Western blot, respectively. The gray value of protein bands was quantified by Image J. **C** The cell viability of MDA-MB-231 cells over-expressing LAMC2 was detected by CCK-8. **D**, **E** The DNA synthesis rate of LAMC2 knockdown cell was detected by EdU assay. **F** The wound healing assay was performed in MDA-MB-231 cells over-expressing of LAMC2. **G**, **H** The migration and matrigel invasion of MDA-MB-231 cells over-expressing LAMC2 were detected by transwell assay. Results were expressed as mean ± SD. Statistical analyses were conducted using a Student’s *t*-test. *P < 0.05; **P < 0.01; ***P < 0.001

To knockdown the expression of LAMC2, two different nucleotide sequences were designed for shRNAs. The knockdown efficiency of LAMC2 was detected by qRT-PCR and western blot using MDA-MB-231 cells, respectively (Fig. 2A, B). To examine the effects of LAMC2 on cell proliferation, a CCK-8 assay was performed in MDA-MB-231 cells after knockdown of LAMC2 (Fig. 2C). There was a significant decrease in LAMC2 inhibited cells compared to control. An EdU incorporation assay confirmed the effects of LAMC2 on the proliferation of TNBC cells (Fig. 2D, E). Besides, the wound healing assay showed that the average migration rate of the LAMC2 knockdown cells was significantly decreased compared to the control group at both 24 h and 48 h after scraping (Fig. 2F). The Transwell assays revealed a significant decrease in migration and matrigel invasion of MDA-MB-231 cells with LAMC2 knockdown compared to the control (Fig. 2G, H). Overall, the data suggested that knockdown of LAMC2 remarkably inhibited cell growth and motility.

### LAMC2 greatly promoted cell proliferation and migration potential of TNBC cells in vitro and in vivo

**Fig. 3:**
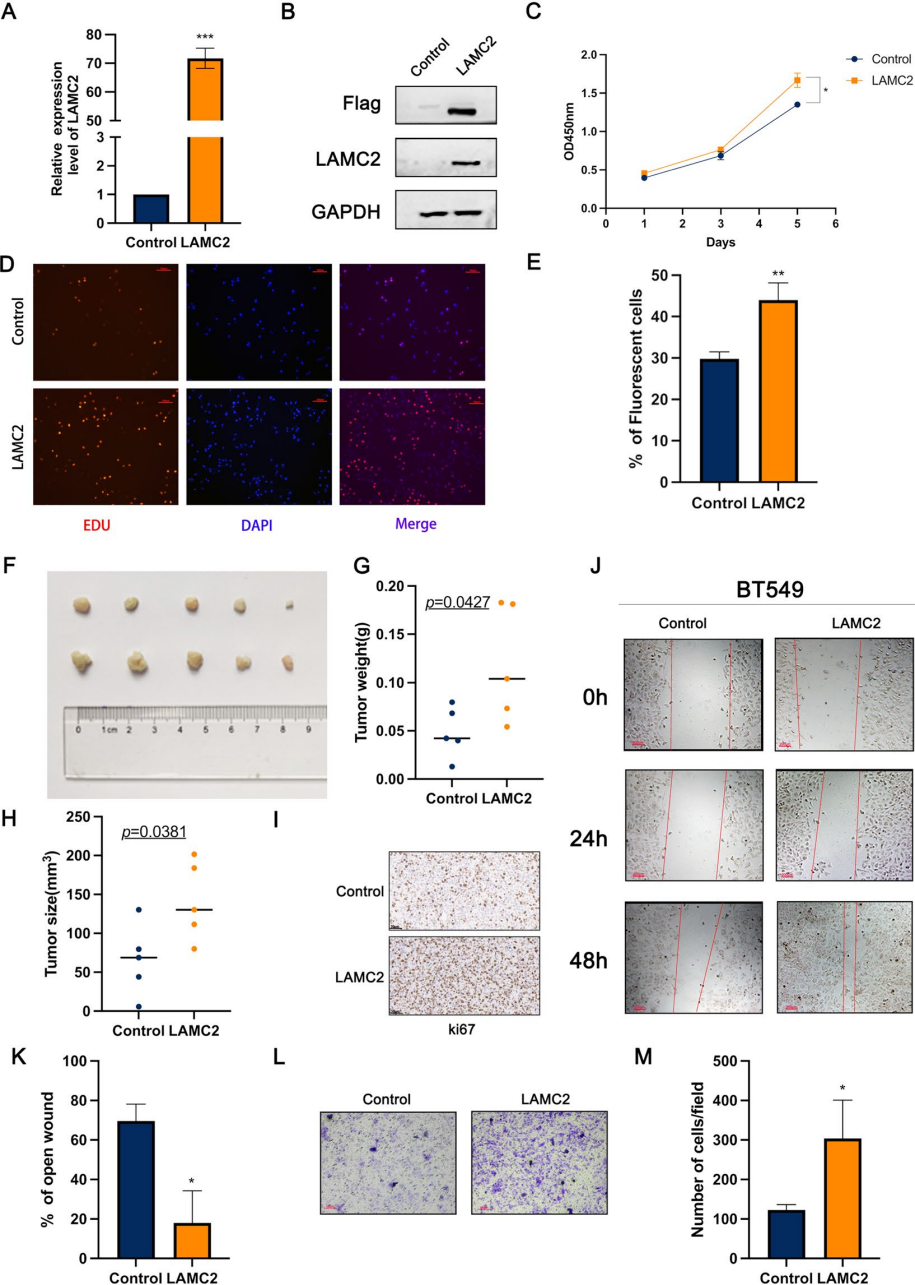
Over-expression of LAMC2 promoted TNBC cells growth and migration in vitro and in vivo. **A**, **B** The over-expression of LAMC2 in BT549 cell lines was detected by qRT-PCR and Western blot, respectively. **C** The proliferation of TNBC cells over-expressing LAMC2 was detected by CCK-8. **D**, **E** The DNA synthesis rate of LAMC2 over-expressing cell was detected by EdU assay. **F** Images of derived tumors from xenograft mice. **G**, **H** Tumor weight and size were scored (n = 5). Results were expressed as mean ± SD. Statistical analyses were conducted using a Student’s *t*-test. *P* < 0.05 was considered as a significant difference. I. Representative images of IHC for ki67 in tumor and control. Scale, 50 µm. **J**, **K** The wound healing assay was performed in BT549 after over-expressing LAMC2. **L**, **M** The migration of TNBC cells over-expressing LAMC2 was detected by transwell assay. Results were expressed as mean ± SD. Statistical analyses were conducted using a Student’s *t*-test. *P < 0.05

To further investigate the function of LAMC2 on tumor progression, LAMC2 was over-expressed in BT549 cells and the expression efficiency was evaluated using qRT-PCR and western blot, respectively (Fig. 3A, B). Subsequently, the effect of LAMC2 on cell proliferation and migration were conducted through CCK-8, EdU assay, wound healing assay and transwell. The results showed that proliferation abilities of LAMC2 over-expressed cells were enhanced compared to the control cells as detected by CCK-8 (Fig. 3C). Moreover, EdU incorporation assay was also used as a further study to determine the effects of LAMC2 on the proliferation of TNBC cells (Fig. 3D, E). To investigate the impact of LAMC2 on growth of TNBC cells in vivo, a tumourigenicity assay was performed. The data showed that LAMC2-overexpressing cells had higher tumorigenic activity compared to control cells (Fig. 3F). The weight and size of tumors were substantially larger in the LAMC2-overexpressing group than those in the control group (Fig. 3G, H). The Ki67 staining of the tumor sections demonstrated significantly higher proliferative activity in the LAMC2 group than in the control group (Fig. 3I). In addition, the wound healing assay, migration and invasion assay demonstrated that ectopic expression of LAMC2 significantly promoted migration in TNBC cells (Fig. 3J–M, Supplemental Figure S1). These results further confirmed that LAMC2 enhanced the cell growth and migration of TNBC in vitro and in vivo.

### CD44 silencing impeded LAMC2 induced the migration of TNBC cells

**Fig. 4:**
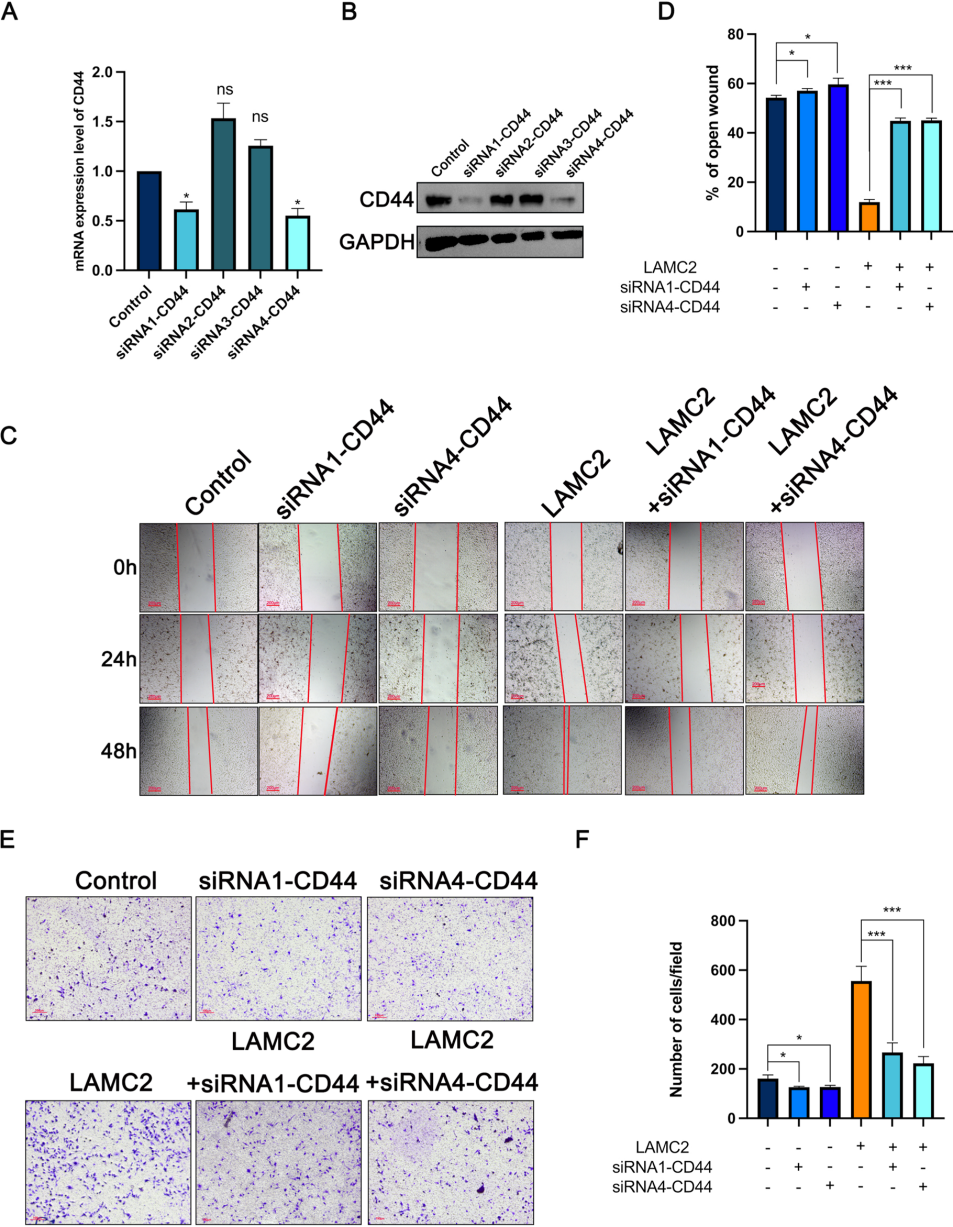
Depletion of CD44 largely reversed the roles of LAMC2 in modulating TNBC cellular migration. **A**, **B** qRT-PCR and western blot analysis showed the expression of siRNA-CD44 in MDA-MB-231 cells, respectively. **C**, **D** The cell migration of TNBC cell over-expressing LAMC2 after treated with siRNA-CD44 was examined by wound healing assay. **E**, **F** The cell migration of TNBC cell over-expressing LAMC2 after treatment with siRNA-CD44 was examined by transwell assay. Results were expressed as mean ± SD. Statistical analyses were conducted using a Student’s* t*-test. *P < 0.05; **P < 0.01; ***P < 0.001. ns: no significance

Given that CD44 was identified to directly interact with LAMC2 on the membrane of breast cancer cells, we further investigated whether CD44 is a critical mediator of the oncogenic roles of LAMC2 in TNBC. Initially, expression efficiency of siRNA-mediated silencing of CD44 were examined by qPCR and western blot, respectively. As shown in Fig. 4A, B, the expression of CD44 in siRNA1-CD44 and siRNA4-CD44 was significantly lower than that in the control and the other two groups. Thus, siRNA1-CD44 and siRNA4-CD44 were selected for use in subsequent experiments. Consistent with a previous study (Nam et al. 2015), the wound healing assay and transwell assay showed that silencing CD44 decreased the migration of TNBC cells (Fig. 4C–F). Notably, depletion of CD44 restored LAMC2-induced promotion of migration. These data demonstrated that LAMC2 exerted oncogenic roles in TNBC cellular growth and migration through up-regulating CD44.

### ZEB1 was a downstream target for LAMC2 in TNBC

**Fig. 5:**
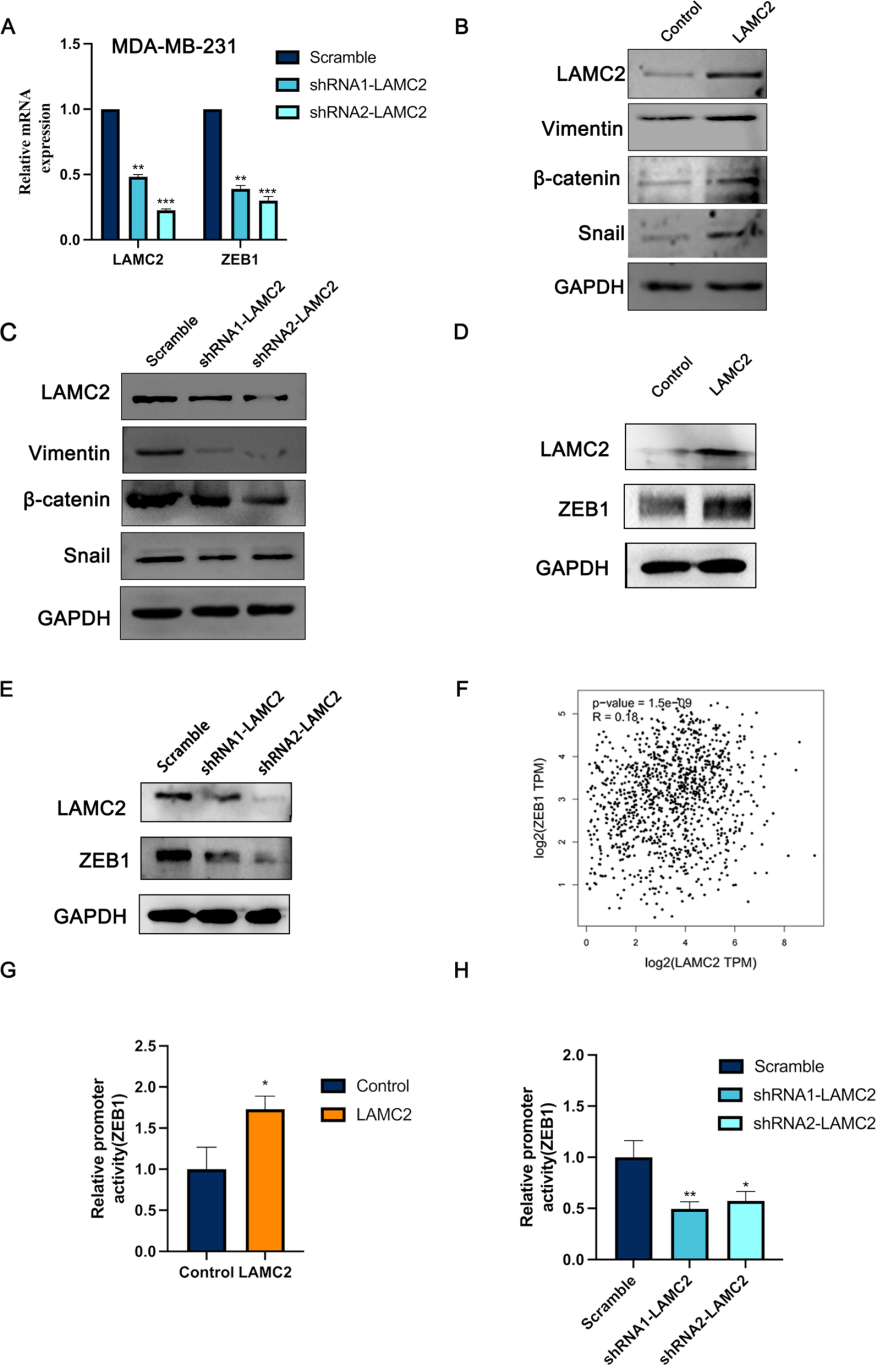
LAMC2 promoted migration through the EMT signaling pathway in TNBC cells. **A** The expression of ZEB1 in MDA-MB-231 cells treated with LAMC2 shRNA or negative controls was examined by qPCR. **B**, **C** The expression of Vimentin, β-catenin and Snail in MDA-MB-231 cells treated with either LAMC2 shRNA or over-expression LAMC2 was examined by western blot. **D**, **E** The expression of ZEB1 in MDA-MB-231 cells treated with either LAMC2 shRNA or over-expression LAMC2 was examined by western blot. **F** The correlation between the mRNA expression levels of LAMC2 and ZEB1 in TNBC samples was examined using the TCGA database. **G**, **H** Quantification of ZEB1 promoter luciferase activity in HEK293T cells transfected with knockdown of LAMC2 or over-expression LAMC2. Results were expressed as mean ± SD. Statistical analyses were conducted using a Student’s *t*-test. *P < 0.05; **P < 0.01; ***P < 0.001

Previous studies have shown that an isoform shift from CD44v to CD44s contributed to EMT and breast cancer progression (Zhang et al. 2019). Therefore, we hypothesized that LAMC2 may promote migration through regulation of EMT. To examine this hypothesis, we investigated the mRNA expression of EMT-related genes in MDA-MB-231 cells treated with LAMC2 shRNA or negative controls. The data showed that knockdown of LAMC2 in TNBC cell lines resulted in reduced expression of β-catenin, Snail, slug, Twist, ZEB1 and ZEB2 (Fig. 5A and Supplemental Figure S2). Moreover, the western blot showed that the protein expression of Vimentin, β-catenin and Snail were positively correlated with LAMC2 expression (Fig. 5B, C). Since LAMC2 exerted oncogenic roles in TNBC through up-regulating CD44, we evaluated the effect of CD44 on the TNBC associated EMT process. As expected, the western blot showed that depletion of CD44 restored LAMC2-induced promotion of β-catenin, Snail and Vimentin (Supplemental Figure S3). Notably, ectopic expression of LAMC2 significantly promoted the expression of ZEB1, whereas knockdown of LAMC2 remarkably inhibited the expression of ZEB1 (Fig. 5D, E). As an important EMT-inducing transcription factors, accumulating evidence indicates that ZEB1 is highly expressed in TNBC and involved in tumorigenicity and metastasis by promoting cell motility and stemness (Krebs et al. 2017; Prodhomme et al. 2021). To further determine the clinical relevance of LAMC2 and ZEB1, the association between LAMC2 and ZEB1 in cancer tissues from TNBC patients was examined using the TCGA database. The results showed that LAMC2 was positively correlated with ZEB1 (P = 1.5e−9) (Fig. 5F). To assay the targeting association of ZEB1 with LAMC2, a luciferase reporter assay was performed. The results showed that luciferase activity of the ZEB1 promoter was greatly decreased by knockdown of LAMC2, whereas it was increased by over-expressing LAMC2 (Fig. 5G, H), which indicated that ZEB1 was one of the targets of LAMC2. Overall, these results suggested that knockdown of LAMC2 remarkably inhibited cell motility via EMT and ZEB1 was a downstream target for LAMC2 in TNBC.

### LAMC2 upregulated the expression of ZEB1 through the STAT3 signaling pathway in TNBC cells

**Fig. 6:**
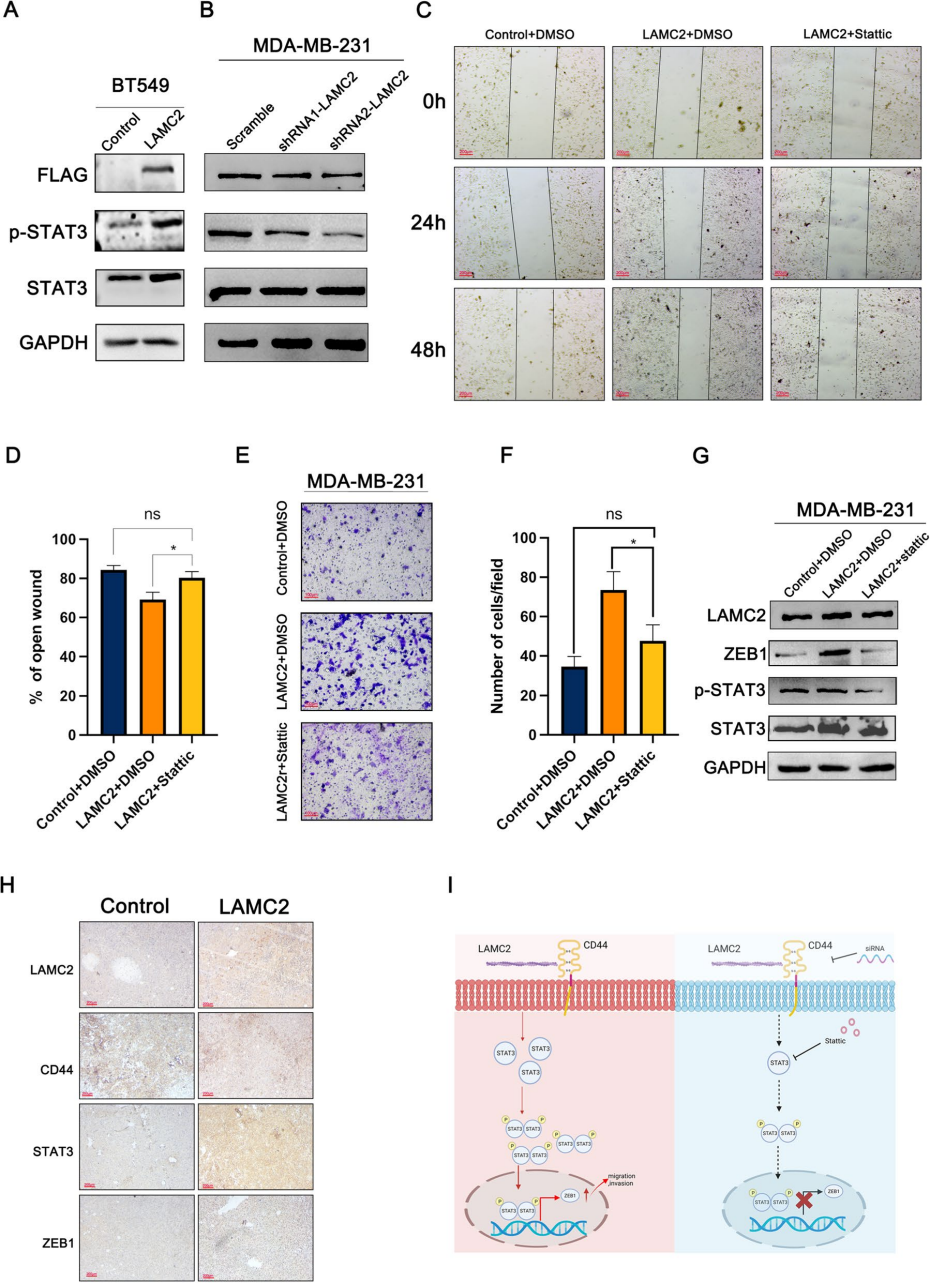
LAMC2 activated STAT3 signaling pathway in TNBC cells. **A**, **B** The protein expression levels of STAT3 and p-STAT3 were examined in TNBC cells either over-expressing LAMC2 or repressing LAMC2. **C**, **D** The cell migration of TNBC cell over-expressing LAMC2 after treatment with Stattic were examined by wound healing assay. **E**, **F** The cell migration of TNBC cells over-expressing LAMC2 after treatment with Stattic was examined by transwell assay. **G** The expression of STAT3, p-STAT3 and ZEB1 was examined in LAMC2 over-expressing cells treated with Stattic by western blot. **H** The expression of LAMC2, CD44, ZEB1 and STAT3 in LAMC2 over-expressing tumor tissues of mice was evaluated by IHC. **I** A cartoon depiction of the effect of LAMC2/CD44/STAT3/ZEB1 signaling pathway was shown. Results were expressed as mean ± SD. Statistical analyses were conducted using a Student’s *t*-test. *P < 0.05. ns: no significance

It has been reported that STAT3 directly mediated EMT progression and regulated ZEB1 expression in colorectal cancer (Xiong et al. 2012). Thus, we performed western blot to examine the protein expression levels of STAT3 and STAT3 phosphorylation (p-STAT3). The results showed that the expression levels of p-STAT3 were elevated in response to ectopic expression of LAMC2, whereas p-STAT3 were decreased in LAMC2 repressing cells (Fig. 6A, B). Furthermore, we examined whether the observed LAMC2-mediated increase in migration was dependent on STAT3 activation by wound healing assay and transwell assay. As expected, the enhanced effect of LAMC2 over-expression on cell migration was inhibited by STAT3 inhibitor Stattic treatment (Fig. 6C–F). Consistently, Stattic treatment inhibited p-STAT3 and ZEB1 expression in LAMC2 over-expressing cells (Fig. 6G). In addition, we verified LAMC2, CD44, STAT3 and ZEB1 expression in LAMC2 over-expressing tumor tissues of mice by IHC. The results showed that LAMC2/STAT3/ZEB1 were increased in the LAMC2 over-expressing group when compared to control group (Fig. 6H). Taken together, these data strongly supported the notion that LAMC2 targeted ZEB1 via activating CD44/STAT3 signaling pathway to promote TNBC proliferation and migration. A diagram depicting the effect of LAMC2/CD44/STAT3/ZEB1 in TNBC was shown in Fig. 6I.

## Discussion

Laminins are the main component of the basement membrane, composing of α, β and γ chains. A growing number of research reported that Laminins are involved in various biological processes, including cellular phenotype maintenance, adhesion, migration, growth and differentiation (Yao 2017). Recently, lamininγ2 (LAMC2) has been found to be highly expressed in a wide range of cancers. For instance, LAMC2 promoted Akt-Ser473 phosphorylation to enhance cell migration and invasion in pancreatic cancer cells (Wang et al. 2020), implicating that LAMC2 is a promising biomarker and plays an oncogenic role in tumor cells. However, LAMC2 also acts as a tumor suppressor. In hepatocellular carcinoma, LAMC2 has been found to be regulated by miR-548c-3p and inhibited EMT (Jin et al. 2020). High-throughput sequencing results showed that miR338-5p/3p targeted LAMC2 to suppress invasion in salivary adenoid cystic carcinoma cells (Wang et al. 2018). Accordingly, the function of LAMC2 varies depending on the different cancer cell types. Herein, we found that LAMC2 expression was highly increased in TNBC and associated with a poor prognosis for TNBC patients. Moreover, over-expression of LAMC2 greatly promoted the cell proliferation and migration potential of TNBC cells in vitro and in vivo*,* suggesting that LAMC2 exerts as an oncogene in TNBC.

Previous studies have revealed that LAMC2 promoted the migration of TNBC cells by directly interacting with CD44 (Sato et al. 2015). Consistently, we found that depletion of CD44 largely reversed the roles of LAMC2 in modulating TNBC cellular growth and migration. CD44 is a non-kinase transmembrane receptor and involved in modulating cellular signaling by forming co-receptor complexes (Ponta et al. 2003). A switch in expression from the variable CD44v isoforms to the standard CD44s isoform through alternative splicing resulted in accelerating both EMT and breast cancer progression (Brown et al. 2011). Due to lacking of all variant exons, the full-length standard CD44 (CD44s) protein is around 85kD. However, besides standard exons, CD44v contains variable exons, resulting in the molecular size ranging form 100–250 kD (Hu et al. 2017; Guo et al. 2021). In our study, CD44s (85 kD), but not CD44v is involved in LAMC2 induced cell proliferation and migration (Fig. 4B). Accumulating evidences indicated that EMT plays a vital role in cancer progression of metastasis. For instance, Okada et al*.* found that LAMC2 promoted cellular migration and invasion in pancreatic cancer cells through regulation of EMT and ATP-binding cassette (ABC) transporter, resulting in a poor prognosis and gemcitabine sensitivity in patients with pancreatic cancer (Okada et al. 2021). In lung cancer, LAMC2 promoted migration and invasion via EMT that was integrin β1- and ZEB1-dependent (Moon et al. 2015). Moreover, the expression levels of LAMC2 in invasive colorectal carcinoma cells were directly regulated by ZEB1 and activated β-catenin (Hlubek et al. 2001; Sanchez-Tillo et al. 2011). Similarly, our results showed that LAMC2 may enhance migration through the induction of EMT and ZEB1 was a downstream target for LAMC2 in TNBC.

Furthermore, it has been reported that STAT3 directly mediated EMT progression and regulated ZEB1 expression in colorectal cancer (Xiong et al. 2012). The activation of STAT3 was often associated with the growth, survival and invasion of breast cancer cells (Gritsko et al. 2006; Hedvat et al. 2009). On the other hand, inhibition of STAT3 signaling by shRNA or the use of STAT3 phosphorylation inhibitors repressed the formation and growth of xenograft tumors in mice as well as the invasive potential of breast cancer cells (Ling and Arlinghaus 2005; Lin et al. 2010). Likewise, we found that LAMC2 promoted STAT3 signaling pathway in TNBC. The enhanced effect of LAMC2 over-expression on cell migration was inhibited by Stattic treatment.

In summary, these findings suggested that higher expression of LAMC2 significantly correlated with poor survival in TNBC cohort. LAMC2 promoted migration capacity of TNBC cell lines via regulating the expression of CD44. Interestingly, LAMC2 targeted ZEB1 and activated the STAT3 signaling pathway and promoted cell migration in TNBC cells. The STAT3 inhibitor Stattic could abolish LAMC2-promoted malignant phenotypes of TNBC cells. Collectively, these results suggested that LAMC2 might act as an oncogene and a potential therapeutic target in TNBC patients.

### Supplementary Information

Supplementary Material 1.

## Data Availability

The original contributions presented in the study are included in the article, and further inquiries can be directed to the corresponding authors.
