# Supplementary material for: Extracellular matrix marker LAMC2 targets ZEB1 to promote TNBC malignancy via up-regulating CD44/STAT3 signaling pathway
Source: Mol Med. 2024 May 17;30:61. doi: 10.1186/s10020-024-00827-6 (PMC11100204; doi:10.1186/s10020-024-00827-6)
Supplement: Supplementary file 1 — Supplementary Material 1. [file 10020_2024_827_MOESM1_ESM.docx]

Supplemental Figure S1


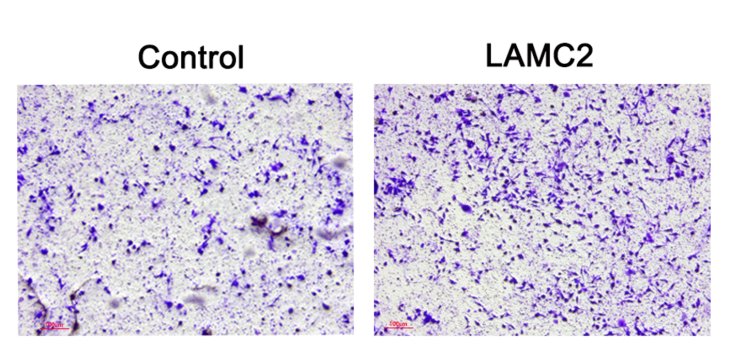


The matrigel invasion of TNBC cells over-expressing of LAMC2 was detected by transwell assay.

Supplemental Figure S2


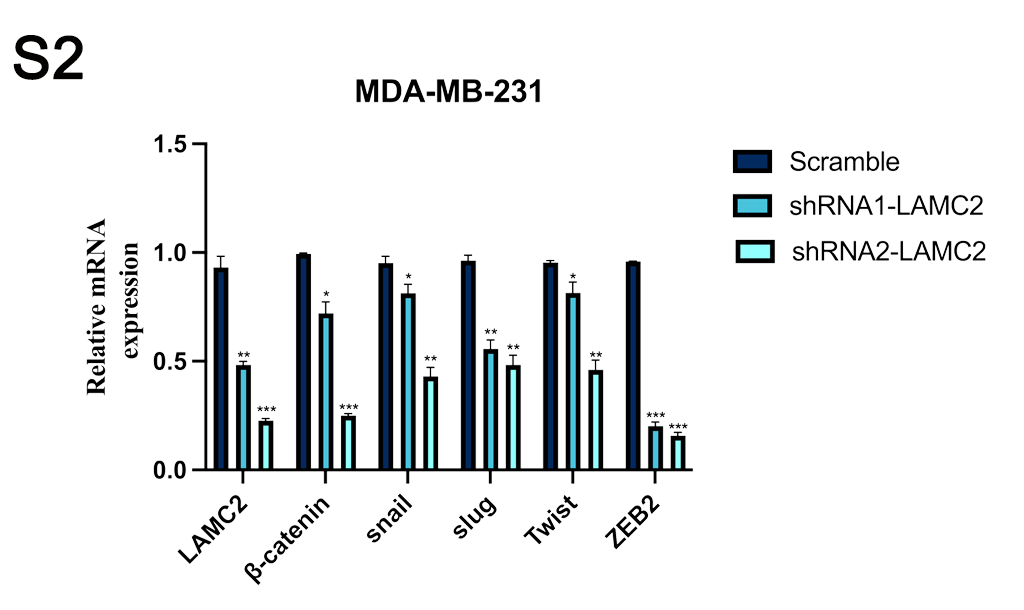


The mRNA expression of β-cadherin, snail, slug, Twist and ZEB2 was examined in TNBC cell lines knockdown of LAMC2. Results were expressed as mean ± SD. *, *P*<0.05; **, *P*<0.01; ***, *P*<0.001.

Supplemental Figure S3


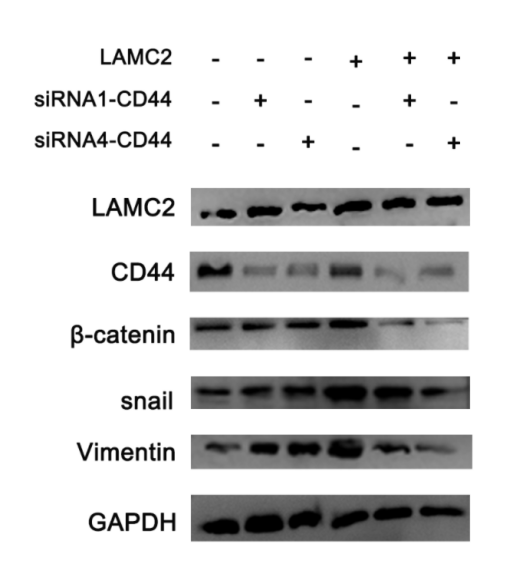


The protein expression of EMT markers were examined in LAMC2-overexpressing cells with depletion of CD44 by western blot.
